# Sequential Therapy in Metastatic Renal Cell Carcinoma

**DOI:** 10.15586/jkcvhl.2016.46

**Published:** 2016-04-05

**Authors:** Bradford R. Hirsch, John M. Burke, Manish Agrawal, Ralph J. Hauke, Thomas E. Hutson, Gury Doshi, Mark T. Fleming, Nicholas J. Vogelzang

**Affiliations:** US Oncology Research, Houston, TX, USA

**Keywords:** kidney cancer, renal cell carcinoma, sequential therapy

## Abstract

The treatment of metastatic renal cell carcinoma (mRCC) has changed dramatically in the past decade. As the number of available agents, and related volume of research, has grown, it is increasingly complex to know how to optimally treat patients. The authors are practicing medical oncologists at the US Oncology Network, the largest community-based network of oncology providers in the country, and represent the leadership of the Network’s Genitourinary Research Committee. We outline our thought process in approaching sequential therapy of mRCC and the use of real-world data to inform our approach. We also highlight the evolving literature that will impact practicing oncologists in the near future.

## Introduction

The treatment of metastatic renal cell carcinoma (mRCC) has changed dramatically in the past decade. Between 2005 and 2012, six new therapies were approved for patients with metastatic disease, in addition to the older therapies of interferon and high-dose interleukin-2 (IL-2) (1). Furthermore, the introduction of new agents is not slowing. In September 2015, two landmark articles on nivolumab and cabozantanib in the *New England Journal of Medicine* advanced the field further (2, 3). Although the new therapies improve outcomes of patients with metastatic disease, there remain as many questions as answers about how best to incorporate the options into treatment algorithms for patients, creating a challenge for practicing oncologists.

To keep up with all of the literature is a daunting task. Guidelines published by groups such as the National Comprehensive Cancer Network (NCCN) help to guide treatment selection (4), yet often the multitude of choices can leave a practitioner uncertain as to which agent to select for a patient. There are also alternative guidelines such as those released by the European Association of Urology and the European Society for Medical Oncology (ESMO) (5, 6). As an example, the most recent NCCN guidelines list six therapeutic options for the first-line treatment of mRCC—sunitinib (Sutent, Pfizer), temsirolimus (Torisel, Pfizer), bevacizumab (Avastin, Roche/Genentech), interferon, pazopanib (Votrient, Novartis), high-dose IL-2, and sorafenib (Nexavar, Bayer)—as well as clinical trials and best supportive care. Other guidelines, such as those released in 2014 by ESMO, refer to stronger evidence for some, but are still quite inclusive. Despite the overlap in some of the classes of agents among these options, there is variability in their toxicity and efficacy profiles, so they are not readily interchangeable. Choice of second-line therapy and beyond becomes complex as many agents are available, including axitinib (Inlyta, Pfizer), everolimus (Afinitor, Novartis), sorafenib (Nexavar, Bayer), and nivolumab (Opdivo, Bristol-Meyers Squibb). To further complicate the picture, patients often become sicker as the disease progresses, forcing physicians to incorporate other considerations such as performance status, comorbidities, and preferences regarding end-of-life care into decisions about treatment options; in fact, evidence suggests that only about 50% of patients in the United States receive second-line therapy, as opposed to best supportive care (7). Italian analyses similarly showed that approximately 50% of patients received second-line therapy, and only 13% of patients received third-line therapy (8, 9).

The authors are practicing medical oncologists at the US Oncology Network (USON), the largest community-based network of oncology providers in the country, who treat relatively high volumes of mRCC patients as members of the Genitourinary Research Committee. Herein, we outline our thought process in approaching sequential therapy of mRCC. The discussion represents our present approach and provides a framework upon which new agents and evidence need to be incorporated as they are introduced over the next months and years. Our experience is further advanced by the “real-world data” analyses that occur within the USON, leveraging the data generated by our treatments to provide new insights into how agents perform in routine clinical care. Such real-world data are critically important in order to demonstrate the external validity of the trial data upon which the guidelines are based.

## Standard of care in the first-line therapy

**Figure 1.**
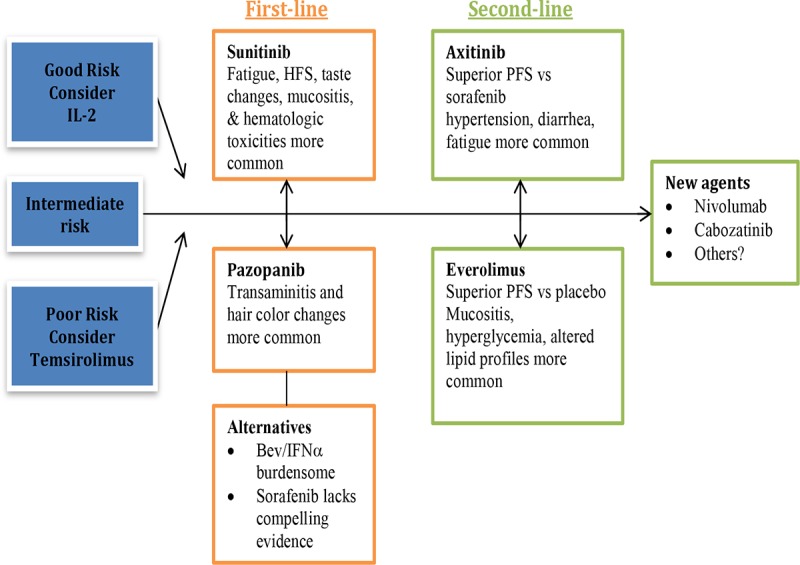
Treatment decision tree for first- and second-line therapy in metastatic renal cell carcinoma based on available and evolving evidence. Bev: bevacizumab; HFS: hand foot syndrome; IFNα: interferon alfa; PFS: progression free survival.

Although there are six treatments listed as first-line options as per the NCCN guidelines, treatment selection becomes more manageable once patients are stratified according to risk category. We recommend risk stratification of patients according to the Memorial Sloan Kettering Cancer Center (MSKCC) criteria (10). As shown in **Table 1**, patients were categorized by five criteria into risk groups: good-risk (0 risk factors), intermediate-risk (1–2), or poor-risk (≥ 3). The Heng criteria were published following the introduction of subsequent agents and can alternatively be used for risk stratification (11). Both frameworks have significant prognostic implications with differences in the median overall survival (OS) in the original MSKCC trial of 19.9 months in the good-risk group compared with 3.9 months in the poor-risk group. In the Heng analysis (post introduction of newer agents), median OS was not reached in the good-risk group, was 27 months in the intermediate-risk group, and was 8.8 months in the poor-risk group. Neither is ideal because they do not fully account for the present era of targeted agents; however, they remain widely used due to their simplicity and the lack of alternative, validated tools. Based on this stratification, patients may be taken down different first- and second-line treatment pathways as shown in **Figure 1**. We outline the logic behind these treatment considerations subsequently.

**Table 1.** Memorial Sloan Kettering Cancer Center (MSKCC) (10) and Heng risk (11) stratification

Variables	Cutoff	MSKCC	Heng
Karnofsky performance status	<80%	X	X
Hemoglobin	<ULN	X	X
Calcium	>10	X	X
Time from diagnosis to treatment	<1 year	X	X
LDH	>1.5× ULN	X	
Platelet count	>ULN		X
Neutrophil count	>ULN		X

ULN: upper limit of normal.

Risk groups are defined as 0 risk factors = favorable, 1–2 risk factors = intermediate, and >2 risk factors = poor.

### Good-risk patients: role of IL-2

For patients who are young, otherwise healthy, and wish to be maximally aggressive, high-dose IL-2 can be considered. In one study, treatment with high-dose IL-2 achieved objective responses in 20% of patients, with complete responses (CRs) in 9% (12). To date, no other agent approved in the treatment of mRCC has achieved similar CR results. The toxicity and intensity of treatment are quite high. Dave deBronkart (e-Patient Dave) gives talks about how his disease was cured by his search of the Internet, identification of IL-2 as an option, and active pursuit of it after his physicians failed to inform him about its availability (13). Patients should be able to similarly weigh the pros and cons of the various treatment options. However, as discussed subsequently, few receive the agent in practice.

### Intermediate-risk patients: role of sunitinib vs pazopanib

About half of patients with mRCC fall into the intermediate-risk category (10). Not taking into account clinical trials, the choice presently is largely between pazopanib and sunitinib for this cohort for the reasons outlined in the “other considerations” section. For these two agents, the landmark trials demonstrated an OS benefit for each agent, as shown in **Table 2**; however, the results cannot be clearly extrapolated to demonstrate a “preferred” agent. Two subsequent studies have informed care. The first is the COMPARZ (Pazopanib vs sunitinib in the treatment of locally advanced and/or metastatic renal cell carcinoma) trial (14). In this trial, 1,110 patients with clear cell mRCC were randomized to pazopanib at 800 mg daily or sunitinib at 50 mg on a 4-week on and a 2-week off schedule. The primary endpoint was progression-free survival (PFS), and the trial was powered for non-inferiority, and not for superiority. The hazard ratio (HR) was 1.05 with a 95% confidence interval (CI) of 0.90 to 1.22. The upper bound of the CI for non-inferiority was predetermined at 1.25, so the trial just met the goal of non-inferiority. There continues to be controversy related to the study design. For instance, true non-inferiority would require both the intention-to-treat and per-protocol approaches to meet the predetermined cut off; however, the CI for the per-protocol had an upper bound of 1.25, equaling the pre-specified cutoff. Despite these limitations, the patient experiences favored pazopanib in terms of fatigue (63% with sunitinib vs 55% with pazopanib), hand-foot syndrome (50% vs 29%), and thrombocytopenia (78% vs 41%). Eleven of 14 Health Related Quality of Life (HRQoL) domains also favored pazopanib. Of note, liver function abnormalities were higher with pazopanib (60% vs 43%). The conclusion of the authors was that pazopanib and sunitinib have similar efficacy as first-line therapy, but the best available toxicity information favors pazopanib.

**Table 2.** Results of landmark(s) trials of FDA-approved agents in the treatment of metastatic renal cell carcinoma in first- and second-line

Agents	Comparator	Study year for OS results	Median PFS, mo	Median OS, mo
**First-line**				
Pazopanib	Placebo	2013 (45)	11.1 vs 2.8*	22.9 vs 20.5
Bev + IFN-α	IFN-α	2010 (21, 24)	10.2 vs 5.4* 8.5 vs 5.2*	23.3 vs 21.3, 18.3 vs 17.4
Sorafenib	IFN-α	2009 (26)	5.7 vs 5.6	Not reported
Sunitinib	IFN-α	2009 (18)	11 vs 5*	26.4 vs 21.8
Temsirolimus**	IFN-α	2007 (17)	5.5 vs 3.1	10.9 vs 7.3*
**Second-line**				
Nivolumab	Everolimus	2015 (2)	4.6 vs 4.4	25.0 vs 19.6*
Axitinib	Sorafenib	2011 (32)	6.7 vs 4.7*	
Everolimus	Placebo	2010 (35)	4.9 vs 1.9*	14.8 vs 14.4

* Statistically significant.

** For poor-risk patients by MSKCC criteria.

Bev: bevacizumab; HD: high dose; IFN-α: interferon alfa; mo: month; OS: overall survival; PFS: progression-free survival.

The second trial of relevance is PISCES (Patient preference study of pazopanib vs sunitinib in advanced or metastatic kidney cancer) (15). A total of 169 patients were randomized to either pazopanib or sunitinib per the dosing approaches outlined above. After 10 weeks on therapy, there was a 2-week washout period, and patients were then switched to the other agent for 10 weeks of further therapy. The primary endpoint was patient preference between agents. Seventy percent preferred pazopanib, 22% sunitinib, and 8% expressed no preference. The main drivers were fatigue and overall quality of life (QOL). The conclusion of the authors was that pazopanib was preferred by patients.

As a result of these two trials, both funded by GlaxoSmithKline, there appears to be a preference for pazopanib in the community and in many academic centers. However, when interpreting the results of COMPARZ and PISCES, one must take into account a few important considerations. First, although not in the product label, many clinicians have subsequently switched from a 4-week on and a 2-week off schedule for sunitinib to a 2-week on and a 1-week off schedule. Early trials have shown improved tolerability with no impact on outcomes (16). It is unknown what impact this change in schedule would have on HRQoL patients shown in the COMPARZ and PISCES trials.

Another key is that the timing of assessments in the trials may have biased in favor of pazopanib by assessing tolerability at the end of the 4 weeks on therapy with sunitinib. This represents the time of maximum toxicity. If taken after the two off weeks, the HRQoL may have been better in the sunitinib arm. Despite these concerns, the data from COMPARZ and PISCES represent the best available information with which to choose between the agents.

### Poor-risk patients: role of temsirolimus

In the same way that IL-2 has a specific indication in the particularly fit patient, temsirolimus has a niche in poor-risk patients. In the phase III trial of the agent, 626 treatment naïve patients were given weekly temsirolimus, temsirolimus plus interferon-alfa, or interferon-alfa alone (17). Monotherapy with temsirolimus was superior to interferon-alfa in terms of OS (10.9 vs 7.3 months, HR, 0.83 [95% CI, 0.58–0.92]). The combination arm was similar to interferon-alfa monotherapy at 8.4 months. A similar superiority for temsirolimus monotherapy was seen in PFS.

No other agents have data specifically supporting their use in the poor-risk space. For example, the pivotal trials for sunitinib and pazopanib included very few poor-risk patients at only 6% and 3%, respectively (18, 19). The subset was, therefore, not large enough to provide compelling data as to their role. There is an ongoing phase II trial comparing temsirolimus with pazopanib, which will further define the role of temsirolimus, as will ongoing real-world analyses (20).

### Other considerations

- Other approved regimens: Although bevacizumab plus interferon-alfa has a category 1 indication in the NCCN guidelines based on the AVOREN and CALGB 90206 (Cancer and Leukemia Group B 90206) results, no trial has demonstrated an OS benefit for the addition of bevacizumab. We do not favor this regimen because of the toxicities of interferon, the need for frequent subcutaneous and intravenous administration of the agents, and the availability of the other orally administered agents discussed above (21–24). Sorafenib does not have a category 1 indication because TARGET (Treatment Approaches in Renal Cancer Global Evaluation Trial) did not show an OS benefit, and the previous phase II did not show a PFS advantage when compared to interferon-alfa (25–27).
- Combinations of agents: Numerous trials have looked at combinations of agents, hoping to demonstrate synergistic effects and tolerable side effects, but they have not been shown to be beneficial. An example is the BEST (BEvacizumab, Sorafenib, and Temsirolimus) trial that compared bevacizumab, bevacizumab plus temsirolimus, bevacizumab plus sorafenib, and sorafenib plus temsirolimus (28). No combination arm showed superiority with a PFS of 8.7 months for monotherapy vs 7.3, 11.3, and 7.7 months for the other arms, respectively. An exception to this lack of benefit to combination therapies can be seen with the recent evidence showing benefit for the combination of lenvatinib and everolimus in the second-line as presented subsequently.

### Real-world practice patterns

The use of data generated in clinical practice to inform treatment considerations has not been fully realized. A recent article compared patients included in the pivotal clinical trials with those treated with sunitinib, sorafenib, pazopanib, and temsirolimus in the community. The “real-world” cohort were part of a joint academic-community registry (29). Overall, 39% of the registry patients would not have met the inclusion and/or exclusion criteria for the relevant pivotal trial used to approve the drug that they received. As an example, among the 438 community patients, those who received tyrosine kinase inhibitors (TKIs) were more likely to have poor-risk disease (7.4% vs 2.9%, *P*<0.001) and less likely to have favorable disease (30.1% vs 43.8%, *P*<0.001) when compared with those in the trials. Those treated with temsirolimus were less likely to have poor-risk disease (10.2% vs 69.4%) when compared with those in the trial, despite poor risk being the indication for the use of the agent.

These findings beg the question of whether those patients treated in the community have similar outcomes and toxicity profiles with those who participate in the clinical trials used to approve a given agent. An abstract presented at the ASCO Genitourinary Meeting in January 2016 described outcomes of USON patients treated with pazopanib or sunitinib in the first-line (30). Median PFS was 9.3 months with pazopanib and 8.3 months with sunitinib when compared with 11 and 11.1 months in the pivotal trials, as shown in **Table 2**. Median OS was also similar between the two agents at 22.3 and 26.3 months in the USON retrospective cohorts, respectively, when compared with 22.9 and 26.4 months in the pivotal trials. These results are reassuring that the outcomes are not dramatically different in our experience overall, or by agent. In the USON retrospective series, adverse events (any grade), including anorexia, skin toxicity, and stomatitis, were significantly less common among pazopanib-treated patients (*P*<0.05), whereas diarrhea, hypertension, nausea, and vomiting were significantly less common with sunitinib (*P*<0.05). Patients treated with sunitinib also appeared to have higher incidence of headache and pain in an extremity although the difference was not statistically significant.

A similar analysis of the community-academic registry as to the rate of adverse events is instructive in showing the tolerability of these agents in practice (7). Of the 466 patients captured in the real-world registry, 57% experienced fatigue that was severe enough to lead to documentation in the chart, 40% vomiting, 34% diarrhea, 33% asthenia, and 21% mucosal inflammation. This demonstrates the frequency of difficulties with tolerance in this patient population. When looking at sunitinib, only 46% of patients remained on full-dose therapy (50 mg) by the end of therapy. Sixteen percent were on a dose of 25 mg or below, calling into question the effectiveness of the therapy being provided in these instances.

In order to optimally manage these patients, we must ensure that effectiveness and tolerability in practice are documented, and disseminated to the treatment community. A recent survey of medical oncology experts representing 11 cancer centers of excellence from around the world assessed variations in treatment approaches (31). The authors found great heterogeneity in decision criteria and were struck by how “differently the available data are interpreted and implemented by experts.” This shows the difficulty in applying the available results from clinical trials to patients in the real world.

### US Oncology Network approach

We recommend either pazopanib or sunitinib as first-line options in the treatment of mRCC. If sunitinib is used, we often use a dose and schedule of 50 mg daily for 2 weeks on and 1 week off based on the data suggesting better tolerability although we acknowledge that the quality of the data is not of the highest level. Although temsirolimus is certainly reasonable in poor-risk patients, the authors are nearly evenly split on its use in practice as the preferred agent. Four use it regularly, one uses it occasionally, and the remaining three do not use it, instead giving pazopanib or sunitinib. IL-2 is rarely used as well. Because of the toxicity and need for hospitalization, we recommend administration only in high-volume centers with considerable experience and in otherwise healthy, younger patients. Among the authors, all consider administering it, yet some have never given it and others do so quite rarely. With the introduction of immunotherapies, the pendulum is likely to swing yet further away.

The hope is that, as real-world data are increasingly refined, they could be used to simplify the treatment algorithm yet further based on the balance of relative value and the validation of findings such as the rate of CR with IL-2 and preferential utility of temsirolimus in the identified niche. To take this one step further, the hope is to personalize treatments for a given patient based on their unique characteristics such as age, comorbidities, and goals of care.

## Standard of care in the second-line

Second-line therapy is changing. Per the NCCN guidelines, if patients receive either IL-2 or temsirolimus in the first-line, they should receive either pazopanib or sunitinib in the second-line. However, for those who receive either pazopanib or sunitinib in the first-line, the choice in the second-line is less straightforward. There is evidence to support both a mammalian target of rapamycin (mTOR) inhibitor and a TKI. The preferred agent has not been clearly demonstrated.

### Axitinib vs everolimus

Axitinib was approved based on the AXIS (Axitinib versus Sorafenib in Advanced Rena Cell Carcinoma) trial in which 723 patients were randomized to axitinib or sorafenib (32). All had received one prior therapy. The median PFS for axitinib was 8.3 vs 5.7 months with sorafenib (HR, 0.665, 95% CI, 0.544–0.812). There was no difference in OS. The most common side effects with axitinib were diarrhea, hypertension, and fatigue. These findings established axitinib as the preferred choice over sorafenib in the second-line setting.

Everolimus was approved based on the RECORD-1 (Renal Cell Cancer Treatment with Oral RAD001 given daily) results in which it was compared to placebo (33–35). In the trial, 410 patients were assigned 2:1 to everolimus or placebo. All had received at least one, if not two, prior treatments with sunitinib and/or sorafenib. The median PFS for everolimus was 4.0 vs 1.9 months with placebo. No OS impact was seen in the trial although the crossover rate was over 90%. It was also clear that there was benefit for everolimus as a third-line agent. The most common adverse events with everolimus included stomatitis, rash, and fatigue. Pneumonitis occurred in 8% of patients.

Because there was not a head-to-head comparison, it is difficult to decide which agent is preferred to date in the second-line. Is a TKI-mTOR strategy preferable to TKI-TKI? The INTORSECT (Investigating Torisel as Second-Line Therapy) trial is somewhat instructive. In this trial of patients previously treated with sunitinib, the PFS results were similar whether a patient was treated with mTOR or TKI in the second-line (4.3 vs 3.9 months, HR, 0.87 [95% CI, 0.71–1.07]); however, OS was inferior in the TKI-mTOR group at 12.3 vs 16.6 months (HR, 1.31 [95% CI, 1.05–163]). The greatest benefit of the TKI-TKI approach was seen in patients who had over 6 months of response to sunitinib in the first-line. A factor in favor of axitinib over everolimus is its ability to produce responses: axitinib produces a response rate of about 20% in the second-line setting, whereas the response rate of everolimus is <5%.

Despite the three large trials discussed (RECORD-1, AXIS, and INTORSECT), there is not a clear treatment of choice in the second-line to date between axitinib and everolimus. INTORSECT looked at temsirolimus, which is not the agent used in the second-line, RECORD-1 compared the agent with placebo, and no trials showed an OS benefit. Although the details are indeed thought provoking, they require assessment in the real world, if not a prospective trial, in order to answer the question of what agent to use. An example of such an analysis can be seen in an article that is presently undergoing peer review, in which the real-world experiences with axitinib and everolimus were compared (36). The results showed that PFS did not differ significantly between everolimus and axitinib (HR, 1.16 [95% CI, 0.73–1.82]). However, axitinib was associated with 17% higher drug costs per month of PFS at $12,467 vs $10,637 (*P*<0.001). This is an example of how real-world data can be used to advance treatment paradigms.

### Evolving second-line literature: roles of nivolumab, cabozantinib, and lenvatinib

Nivolumab is a monoclonal antibody directed at programmed death (PD)-1. Inhibition of PD-1 by nivolumab drives T-cell immunity and has been successful in treating non-small-cell lung cancer and melanoma among other cancer types. In a phase III trial, 821 patients with mRCC with a clear cell component were randomly assigned to receive either nivolumab or everolimus and treated until progression or unacceptable toxicity (2). Nivolumab was administered at a dose of 3 mg/kg intravenously every 2 weeks, and everolimus was administered at a dose of 10 mg orally daily. PFS was not significantly different at 4.6 months with nivolumab vs 4.4 months with everolimus; however, OS was 25.0 and 19.6 months (HR, 0.73 [95% CI, 0.57–0.93]), respectively. Overall response rate (ORR) was 25% with nivolumab vs 5% with everolimus, with similar CR rates of 1% and <1%. Responses were consistent across subgroups of MSKCC risk and PD-L1 status. The rate of grade 3 or 4 toxicity was preferable for nivolumab at 19% vs 37% with everolimus. This trial demonstrated the efficacy of nivolumab in treating mRCC and resulted in the Food and Drug Administration (FDA) approval on November 23, 2015. We predict that nivolumab will be widely used in the treatment of patients with mRCC after failure of a TKI. The role of immune checkpoint inhibitors in the first-line setting is being actively investigated in clinical trials.

Although checkpoint inhibitors garner much of the attention, other promising results from agents are being studied. Cabozantinib (Cometriq, Exelixis) is an oral, small-molecule TKI, a different target profile (37). In the METEOR trial (A Trial of Cabozatinib (XL184) vs Evereolimus in Subjects with Metastatic Renal Cell Carcinoma), 658 patients with metastatic kidney cancer and a clear cell component were randomly assigned to either cabozantinib at 60 mg orally daily or everolimus at 10 mg orally daily (3). Patients had to have received at least one prior TKI. The median PFS was 7.4 with cabozantinib vs 3.8 months with everolimus (HR, 0.58 [95% CI, 0.45–0.75]). The ORR was 21% vs 5% (*P*<0.001). Although there was a trend in favor of improved OS with cabozantinib (HR, 0.67 [95% CI, 0.51–0.89]), it did not meet the predefined boundary for the interim analysis. The data will continue to mature. The main concern is that 60% of patients in the cabozatinib arm required dose reductions vs 25% of patients in the everolimus arm. The discontinuation rates were similar at 9% and 10%, respectively. The conclusion of this study is that, as a therapy for RCC refractory to prior TKI therapy, cabozantinib improves PFS when compared with everolimus. The response rate was similar to axitinib. Whether cabozantinib offers advantages over axitinib, the TKI already FDA approved in the same setting, is unclear. Cabozantinib has not been FDA approved for RCC (although it is approved for medullary thyroid cancer) at the time of this writing.

Finally, a phase II trial was published in *The Lancet* on October 15, 2015, that assessed lenvatinib, everolimus, and lenvatinib/ everolimus in the second-line among 153 mRCC patients (38). Mouse models have shown that the fibroblast growth factor (FGF) may be a mechanism of resistance to TKIs. Lenvatinib has a unique mechanism of action in that it is a potent inhibitor of both VEGF and FGF receptors, potentially providing benefit despite progression on a prior TKI. Lenvatinib use in combination with an mTOR inhibitor showed yet greater activity in these models. The phase III study showed that the combination significantly prolonged PFS when compared with everolimus alone at 14.6 vs 5.5 months (HR, 0.40 [95% CI, 0.24–0.68]) but not with lenvatinib alone at 14.6 vs 7.4 months (HR, 0.66 [95% CI, 0.30–1.1]). Grade 3 or 4 events occurred in 71% of those receiving combination therapy, 79% of lenvatinib-treated patients, and 50% of everolimus-treated patients. No survival data are yet available.

### Real-world practice patterns and the USON pathways

Nivolumab is likely to become the new standard of care in the second-line based on the data showing a preferential toxicity profile, higher response rate, and 5.4-month improvement in OS. Axitinib is still a reasonable option after failure of a prior TKI; however, we predict that the novel mechanism of action of nivolumab, the proven survival advantage (which axitinib does not have), and the general excitement within the oncology community about checkpoint inhibitors will lead to the use of nivolumab. Axitinib may instead be used as third-line. If cabozantinib becomes approved by the FDA, it could also be used as a third-line treatment. Decisions will likely be based on cost, toxicity profile, patient preference, and results of published follow-up. An ongoing trial comparing cabozantinib with sunitinib in the first-line has completed accrual (39).

## Evolving literature and next steps

We predict that there will be extensive study of combinations of agents such as nivolumab with sunitinib, pazopanib, or ipilimumab (40). Although combinations have proven difficult in RCC, the toxicity profile and response of nivolumab may make its use in combination a viable option.

Over the next few years, the role of checkpoint inhibitors will evolve further as other agents are approved, as has occurred with TKI and mTOR inhibitors, and as first-line trials read out. Combinations of checkpoint inhibitors and anti-angiogenic agents will continue to be studied although early trials have shown significant toxicity. A phase 1 trial looking at nivolumab with either sunitinib or pazopanib showed that 73% and 60% of patients experienced grade 3 or 4 toxicities, respectively (41). Dual checkpoint inhibition—such as combining a PD-1 antibody like nivolumab with an alternative immunotherapy—is being studied. Nivolumab with ipilimumab (Yervoy, Bristol Meyers Squibb) was first approved on October 1, 2015, for the treatment of metastatic melanoma. A similar trial in mRCC is ongoing (CheckMate 214). In metastatic melanoma, the combination improved PFS from 4.7 with ipilimumab alone to 8.9 months with the combination (42).

There are other classes of agents in the pipeline as well. An example is the promise of autologous dendritic cell immunotherapy. The ADAPT (Autologous Dendritic Cell Immunotherapy Plus Standard Treatment of Advanced Renal Cell Carcinoma) trial of autologous dendritic cell immunotherapy has completed enrollment of 450 patients as of July 2015 (43). The agent, AGS-003, is being given in conjunction with sunitinib in newly diagnosed patients to assess the effect of a combined approach of TKI and immunotherapy. Data from the phase III IMPRINT (IMA901 in Patients Receiving Sunitinib for Advanced/Metastatic Renal Cell Carcinoma) trial looking at IMA901, a vaccine in combination with sunitinib, were presented at the European Cancer Congress in late 2015 and did not meet the primary endpoint of an extension in OS (44).

Beyond the introduction of new agents, the utility of data and informatics to drive care is promising. Groups such as the USON are establishing systems and approaches that will leverage data from electronic health records, genomic analyses, and other systems to assess risk, improve understanding of the optimal role for specific agents, and provide clinical decision support to enable personalized recommendations. Real-world data could be used to update the analyses and help to further define the roles of available agents. It is likely that the output would be more nuanced and complex than prior approaches; however, it could be supported with available systems.

## Conclusions

Although the mRCC treatment landscape can seem quite complex and overwhelming, the approach to treatment is relatively straightforward. Pazopanib and sunitinib represent the standard-of-care options we prefer for initial therapy in most patients with metastatic disease, with temsirolimus and IL-2 playing a role in limited situations. Nivolumab was recently approved by the FDA and will likely become the preferred second-line therapy. Axitinib is also reasonable to use in the second-line setting. Cabozantinib appears promising, but is not yet FDA approved. We suspect that axitinib and cabozantinib will begin to be used as third-line agents, with everolimus reserved for fourth-line. Sorafenib is also available, but its role at this point has become unclear. Finally, we encourage our patients to consider clinical trials, so that they may have access to the latest discoveries and contribute to finding a cure for kidney cancer.
